# Supplementary material for: Development of Genetically Encoded Fluorescent KSR1-Based Probes to Track Ceramides during Phagocytosis
Source: Int J Mol Sci. 2024 Mar 5;25(5):2996. doi: 10.3390/ijms25052996 (PMC10932182; doi:10.3390/ijms25052996)
Supplement: Supplementary file 1 [file ijms-25-02996-s001.zip › Supplementary_Figures.pdf]

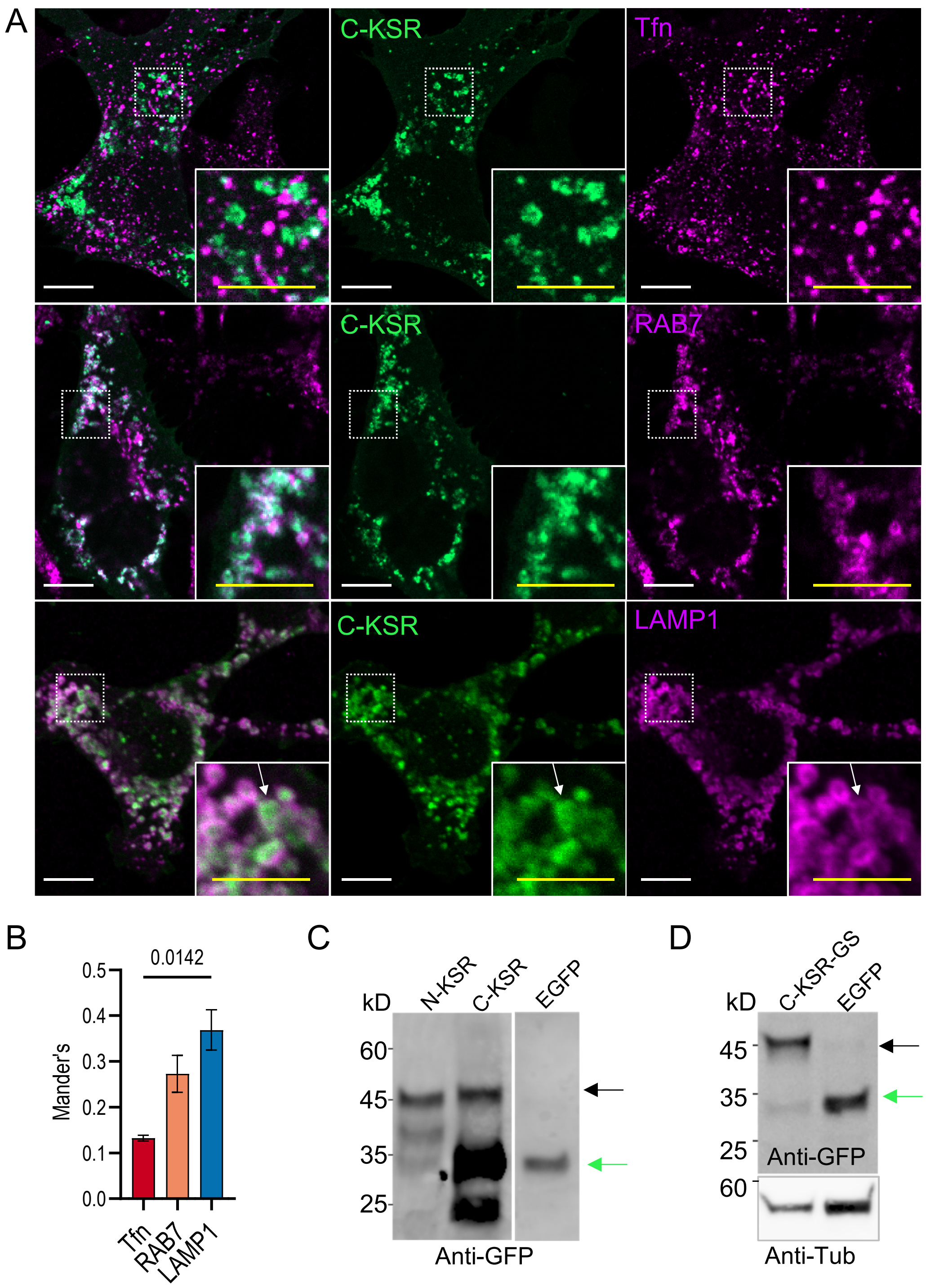

Figure S1. *C-KSR localizes to late endosomes and lysosomes.* **(A)** Confocal images of MEF cells transfected with C-KSR (green) and loaded for 30 min with recycling endosome marker AlexaFluor555-conjugated transferrin (Tfn, 50 µg/ml, magenta top); or immunostained with late endosome marker anti-RAB7 (magenta middle), or lysosomal marker anti-LAMP1 (magenta bottom). The arrow points to an enlarged vesicular structure showing peripheral C-KSR localization with a hollow lumen, that is surrounded still by LAMP1 staining. **(B)** Quantification of co-localization between C-KSR and organelle marker, expressed as Mander's coefficient (n=4/4/14 coverslips for Tfn/RAB7/LAMP1). **(C-D)** Western blot analysis of lysates of MEF cells expressing (EGFP-tagged) N-KSR, C-KSR **(C)** and C-KSR-GS **(D)** or soluble cytosolic EGFP control. Immunolabelled with anti-GFP antibodies (and anti-tubulin in **D**). Black arrow shows the expected size of the intact probe, and the green arrows show the expected size of EGFP alone. Bars are means +/- SEM. White bars = 10 µm, yellow bars = 3 µm

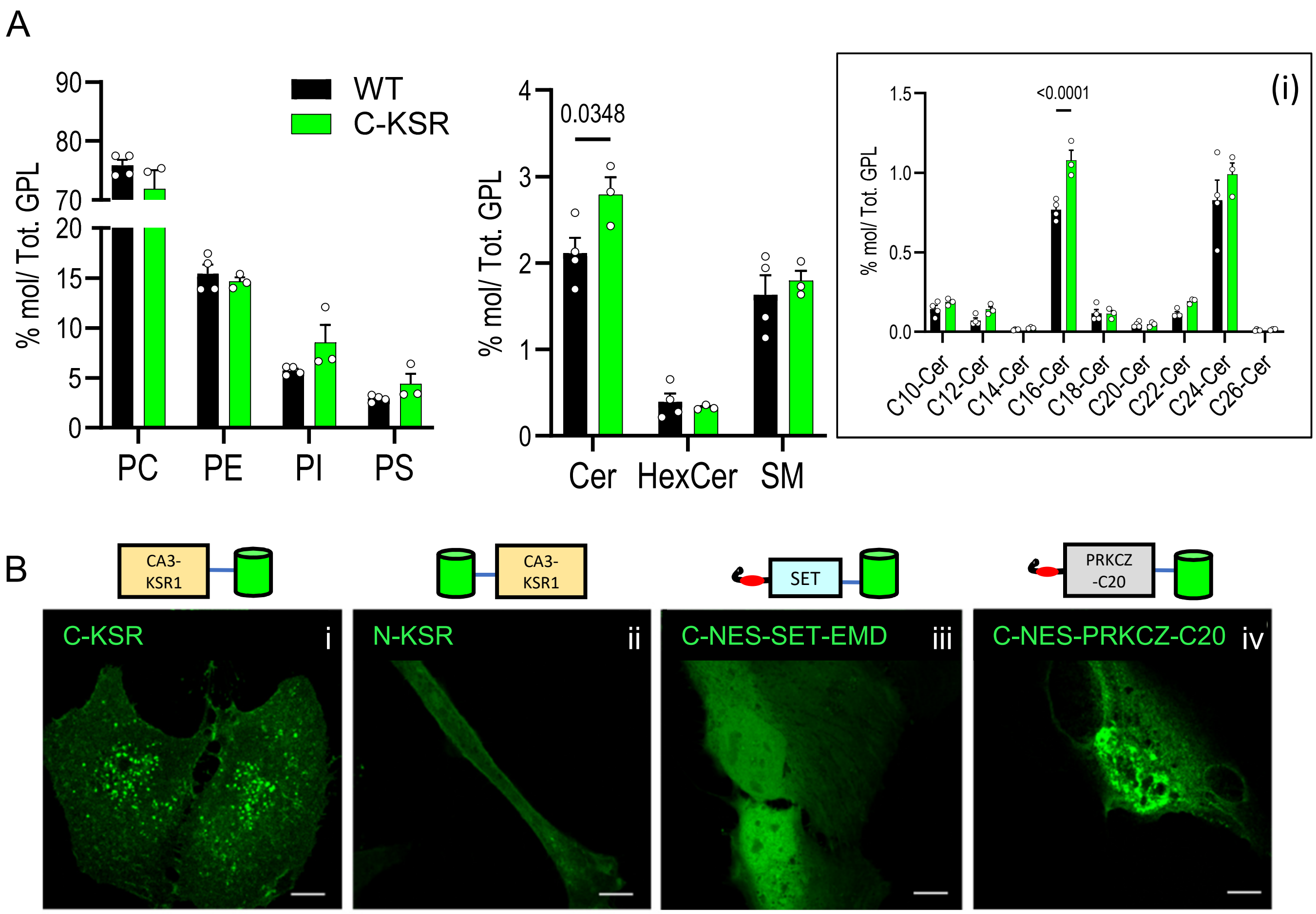

Figure S2. *Lipidomic analysis of C-KSR expression and localization of ceramide probe candidates in HeLa cells.* **(A)** Targeted lipidomic analysis of MEF cells, either wild-type (WT) or stably expressing C-KSR-EGFP (C-KSR). The left panel shows the quantification of the sum total mass of the major glycerophospholipid (GPL) species phosphatidylcholine (PC), phosphatidylethanolamine (PE), phosphoinositide (PI), phosphatidylserine (PS), while the middle panel shows the sum total mass of ceramides (Cer), hexosyl-ceramides (HexCer), and sphingomyelins (SM). Mass values (in fmol) are normalized to the total mass of glycerophospholipids in the same sample (expressed as percent mole, %mol/Tot. GPL). The inset (**i**) on the right shows the quantification of individual ceramide species with carbon chain lengths ranging from C10-Cer to C26-Cer. (n=4/3 WT/C-KSR independent biological samples, see also Supplementary Data S1). **(B)** Confocal images of HeLa cells transiently expressing indicated EGFP-tagged proteins (green): C-KSR (**i**), N-KSR (**ii**) as well as C-NES-SET-EMD (**iii**) and C-NES-PRKCZ-C20 (**iv**) bearing a mitogen-activated protein kinase kinase nuclear export signal (NES). Scale bars, 10 µm. Bars are +SEM.

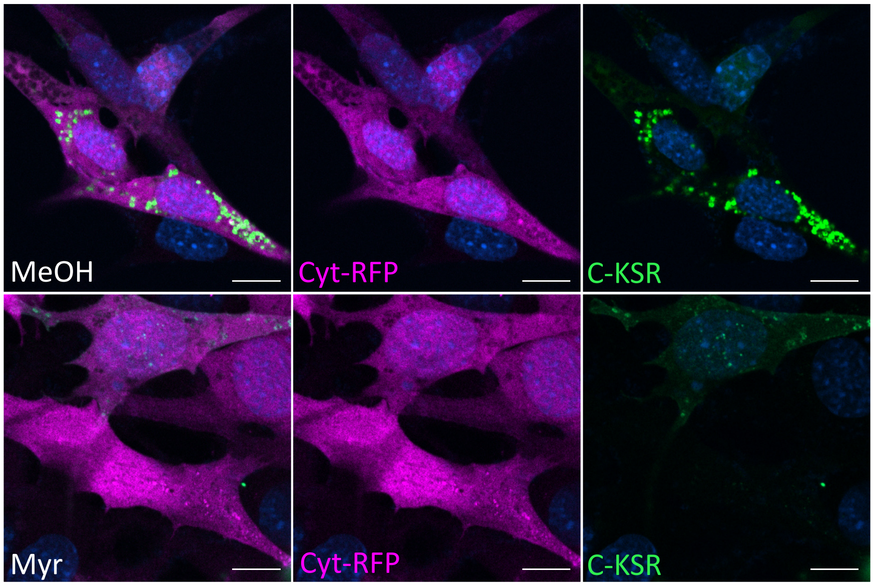

Figure S3. *Effect of myriocin on cytosolic RFP*. Confocal images of MEF cells transiently co-transfected with cytosolic TagRFP (Cyt-RFP, magenta) and C-KSR-EGFP (C-KSR, green) and exposed to 0.5 μM myriocin (Myr) for 3d. White bars = 10 μm.

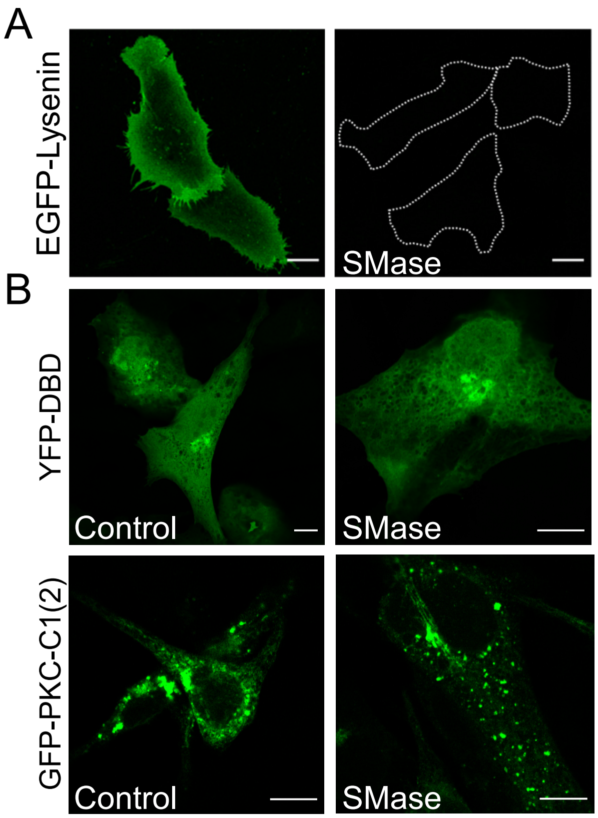

Figure S4. *Other potential ceramide probes do not respond to the local ceramide release at the plasma membrane.* **(A)** Confocal images of fixed MEF cells treated with or without sphingomyelinase (SMase, 0.5U/ml for 30 min in serum-free medium) and stained with a purified sphingomyelin-specific probe EGFP-NT-Lysenin (20 μg/ml). **(B)** Confocal images MEF cells transfected with DAG-specific probes YFP-DBD (top) or PKC-C1(2) (bottom), incubated with 0.5 U/ml SMase for 30 min as above**.** White bars = 10 μm.

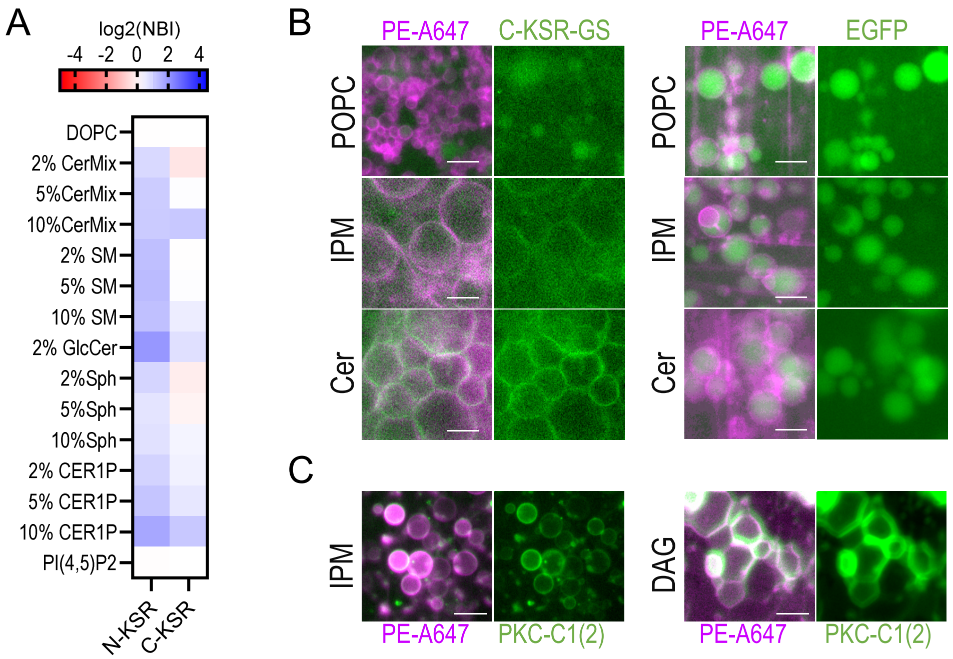

Figure S5. *Liposome microarray (LiMA) analysis of KSR1-based probes*. **(A)** LiMA heatmap showing average binding of purified N-KSR and C-KSR probes to liposomes based on a DOPC backbone. Binding is expressed as log2 transformed normalized binding intensity (NBI) score ratios, computed to the average of DOPC controls. (See Supplementary Data S2 for full dataset). **(B-C)** Representative images of liposomes (containing fluorescent PE-Atto647, magenta) exposed to either C-KSR-GS (B, left, green), negative control EGFP (B, right, green) or positive control PKC-C1(2) (C, green). POPC: control liposomes containing only palmitoyl-oleoyl-PC); IPM: inner plasma membrane mimic liposomes (POPC liposomes containing also PE, PS and cholesterol); Cer: IPM liposomes containing also 10%C18-ceramide. DAG: IPM liposomes containing 5% diacylglycerol (DAG). Bar = 10 µm.

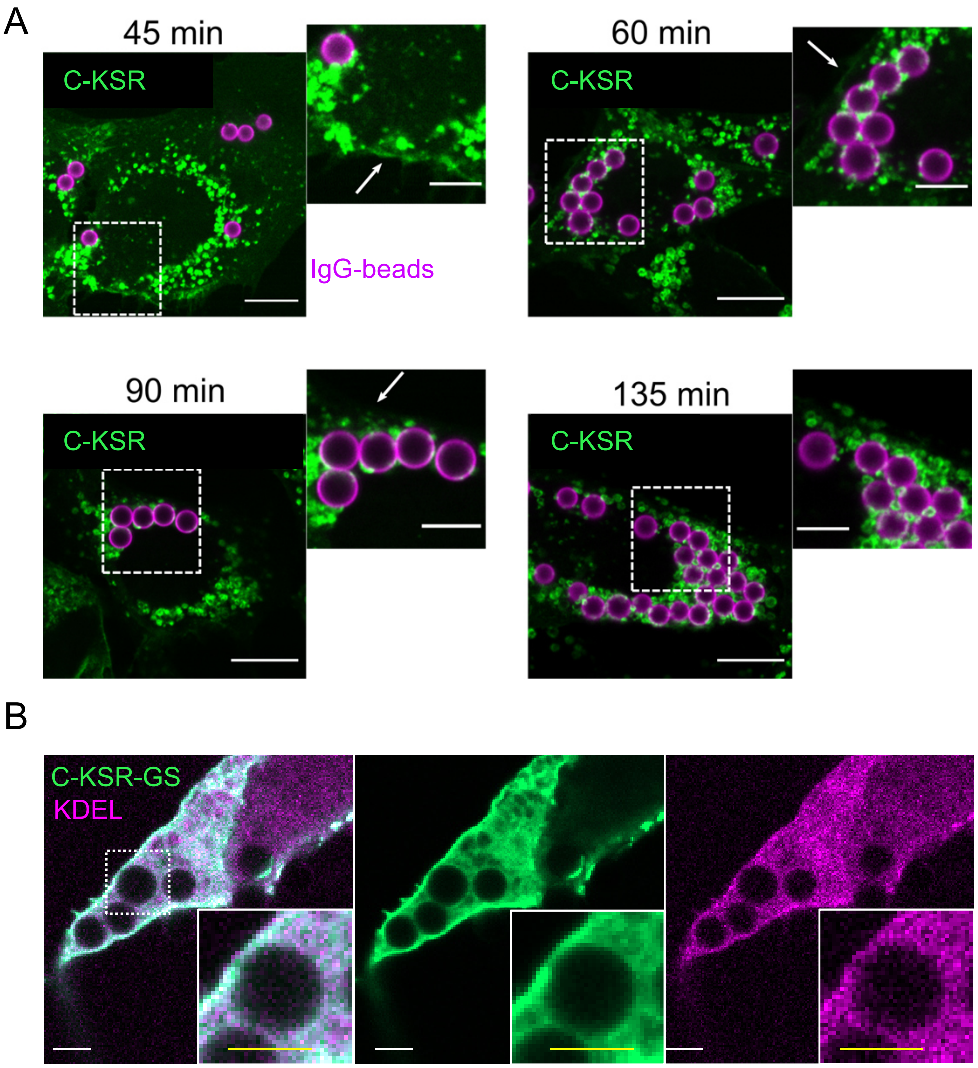

Figure S6. *Behavior of KSR probes during phagocytosis*. **(A)** Confocal images of phagocytic MEFs expressing C-KSR (green) exposed to IgG-Alexa647-coupled beads at 1:10 cell:bead ratio for 45 (upper left), 60 (upper right), 90 (lower left) and 135 (lower right) min. **(B)** Confocal images of phagocytic MEFs co-transfected with C-KSR-GS (green) and ER-targeted soluble TagRFP-KDEL (magenta) and exposed to IgG beads as above for 30 min. White bars = 10 μm, yellow bars = 3 μm.
