# Supplementary material for: Development of Genetically Encoded Fluorescent KSR1-Based Probes to Track Ceramides during Phagocytosis
Source: Int J Mol Sci. 2024 Mar 5;25(5):2996. doi: 10.3390/ijms25052996 (PMC10932182; doi:10.3390/ijms25052996)
Supplement: Supplementary file 1 [file ijms-25-02996-s001.zip › Supplementary_Materials.pdf]

Supplementary Materials

List of Supplementary Materials:

1. Supplementary Figures (separate file):
   - Figures S1 to S6 (file name: Supplementary_Figures.pdf)

2. Supplementary Tables: (below)
   a) **Supplementary Table S1**: List of lipids used in this study.
   b) **Supplementary Table S2**: List of plasmids used and generated in this study.
   c) **Supplementary Table S3**: List of primers used in this study.
   d) **Supplementary Table S4**: Lipid detection parameters for lipidomic analysis

3. Supplementary Data (separate files):
   a) **Supplementary Data S1**: Lipidomic data of wild-type (WT) MEF, or MEF cells stably expressing C-KSR-EGFP cells treated with myriocin, palmitate, sphingomyelinase (worksheets tabs T1 to T3); or MEF cells stably expressing C-KSR-EGFP, transfected with FCGR2A-c-myc and phagocytosing for 0, 30 and 135 min (worksheets T4-T6). Lipid class nomenclature and grouping are summarized in worksheet T7.
   (file name: Supplementary_Data_S1.xlsx)

   b) **Supplementary Data S2**: Liposome microarray quantification using purified C-KSR-sfGFP and N-KSR-sfGFP on DOPC liposomes (worksheet tab T1), HEK lysates of C-KSR-GS-EGFP and N-KSR-EGFP on DOPC liposomes (worksheet tab T2), and HEK lysates of C-KSR-GS-EGFP, EGFP and PKC-C1(2) on IPM liposomes (worksheet tab T3). DOPC: dioleyl-phosphatidylcholine; IPM: inner plasma membrane mimic.
   (file name: Supplementary_Data_S2.xlsx)

Supplementary Table S1. List of lipids used in liposome microarray analysis.

Lipid Catalog Name	Lipid Abbreviation	Symbols/Abbreviations/Other names	Source	Catalog#
1,2-dioleoyl-sn-glycero-3-phosphate (sodium salt)	DOPA	DOPA, 18:1 PA, phosphatidic acid	Avanti Polar Lipids	840875P
1,2-dioleoyl-sn-glycero-3-phospho-(1'-myo-inositol-4',5'-bisphosphate) (ammonium salt)	PI(4,5)P2	DOPI(4,5)P2, PIP2[4',5'](18:1(9Z)/18:1(9Z)) PI(4,5)P2	Avanti Polar Lipids	850155P
1,2-dioleoyl-sn-glycero-3-phosphocholine	DOPC	18:1 (Δ9-Cis) PC (DOPC) phosphatidylcholine	Avanti Polar Lipids	850375P
1,2-dioleoyl-sn-glycero-3-phosphoethanolamine	PE-Atto647	DOPE-Atto647 phosphatidylethanolamine	Atto Tec	AD 647N-161
1,2-dioleoyl-sn-glycero-3-phosphoethanolamine-N-[methoxy(polyethylene glycol)-350] (ammonium salt)	PE-PEG350	PE-PEG350 (18:1) phosphatidylethanolamine	Avanti Polar Lipids	880430O
1,2-dioleoyl-sn-glycero-3-phospho-L-serine (sodium salt)	DOPS	18:1 PS (DOPS), phosphatidylserine	Avanti Polar Lipids	840035P
1-2-dioleoyl-sn-glycerol	DAG	18:1 DG, diacylglycerol	Avanti Polar Lipids	800811O
1-palmitoyl-2-oleoyl-glycero-3-phosphocholine	POPC	6:0-18:1 PC (POPC), phosphatidylcholine	Avanti Polar Lipids	850457P
1-palmitoyl-2-oleoyl-sn-glycero-3-phosphoethanolamine	POPE	16:0-18:1 PE, POPE, phosphatidylethanolamine	Avanti Polar Lipids	850757P
Ceramide from bovine spinal cord[1]	CER	CerMix, bovine ceramide mixture	MilliporeSigma	22244
cholesterol	Chol	3β-Hydroxy-5-cholestene, 5-Cholesten-3β-ol, cholesterol	MilliporeSigma	C8667
D-erythro-sphingosine	Sph	Sphingosine (d18:1)	Avanti Polar Lipids	860490P
D-erythro-sphingosine-1-phosphate	S1P	Sphingosine-1-Phosphate (d18:1)	Avanti Polar Lipids	860492P
D-glucosyl-ß-1,1'-N-heptadecanoyl-D-erythro-sphingosine	GlcCer	glucosyl-ceramide, C17 Glucosyl(ß) Ceramide (d18:1/17:0)	Avanti Polar Lipids	860569P
N-(hexadecanoyl)-sphing-4-enine-1-phosphocholine	SM	16:0 SM, C16-sphingomyelin, Egg SM	Avanti Polar Lipids	860061P
N-palmitoyl-ceramide-1-phosphate (ammonium salt)	Cer1P	C1P, C16-ceramide-1-phosphate (d18:1/16:0)	Avanti Polar Lipids	860533P
N-stearoyl-D-erythro-sphinganine	DHCer	C18 Dihydroceramide (d18:0/18:0)	Avanti Polar Lipids	860627P
N-stearoyl-D-erythro-sphingosine	C18Cer	C18 Ceramide (d18:1/18:0)	Avanti Polar Lipids	860518P

1. *Made from hydrolysis of total bovine brain sphingomyelins. Major components estimated to be 30% C18-ceramide 35% C24-dihydroceramide in [66]*

Supplementary Table S2. List of plasmids used in the study.

Name (short name)	Vector	Insert	Source
pKSR1-CA3-EGFP (C-KSR)	pEGFP-N1	Human KSR1 CA3 domain (aa317-400)	This study Addgene #217753
pKSR1-CA3-mRFP1 (C-KSR)	pmRFP1-N1	Human KSR1 CA3 domain (aa317-400)	This study Addgene #217754
pEGFP-KSR1-CA3 (N-KSR)	pEGFP-C1	Human KSR1 CA3 domain (aa317-400)	This study Addgene #217755
p2X-KSR1-CA3-EGFP (2x-C-KSR)	pEGFP-N1	2x Human KSR1 (aa317-400) fragments in tandem	This study Addgene #217756
pKSR1-CA3-GS-EGFP (C-KSR-GS)	pKSR1-EGFP	GGSSGGGGA flexible linker between the Human KSR1 CA3 domain (aa317-400) and EGFP	This study Addgene #217757

pKSR1-CA3-GS-mRFP1 (C-KSR-GS)	pKSR1-mRFP1	GGSSGGGGA flexible linker between the Human KSR1 CA3 domain (aa317-400) and mRFP1	This study Addgene #217758
pPRKCZ-C1-EGFP (C-PRKCZ-C1)	pEGFP-N1	Human PRKCZ C1 domain (aa123-193)	This study Addgene #217759
pEGFP-PRKCZ-C1 (N-PRKCZ-C1)	pEGFP-C1	Human PRKCZ C1 domain (aa123-193)	This study Addgene #217760
pPRKCZ-C20-EGFP (C-PRKCZ-C20)	pEGFP-N1	20 kD C-terminal domain (C20) of human PRKCZ (aa405-646)	This study Addgene #217761
pEGFP-PRKCZ-C20 (N-PRKCZ-C20)	pEGFP-C1	20 kD C-terminal domain (C20) of human PRKCZ (aa405-646)	This study Addgene #217762
pNES-PRKCZ-C20-EGFP (C-NES-PRKCZ-C20)	pPRKCZ-C20-EGFP	Nuclear export signal from MAPKK preceding the 20 kD C-terminal domain (C20) of human PRKCZ (aa405-646)	This study Addgene #217763
pSET-EMD-EGFP (C-SET-EMD)	pEGFP-N1	Human SET earmuff domain (EMD) (aa70-226)	This study Addgene #217764
pEGFP-SET-EMD (N-SET-EMD)	pEGFP-C1	Human SET earmuff domain (EMD) (aa70-226)	This study Addgene #217765
pNES-SET-EMD-EGFP (C-NES-SET-EMD)	pSET-EMD-EGFP	Nuclear export signal from MAPKK preceding the Human SET earmuff domain (EMD) (aa70-226)	This study Addgene #217770
pETM11-His6-SUMO3-sfGFP		His6-SUMO3-sfGFP	Gavin laboratory [61]
pETM11-SUMO3-EGFP-sfGFP	pETM11-His6-SUMO3-sfGFP	Super-folder-GFP (sfGFP)	Gavin laboratory [61]
pETM11-SUMO3-KSR1-CA3-sfGFP (C-KSR)	pETM11-His6-SUMO3-sfGFP	Human KSR1 CA3 domain (aa317-400)	This study Addgene #217766
pETM11-SUMO3-sfGFP-KSR1-CA3 (N-KSR)	pETM11-His6-SUMO3-sfGFP	Human KSR1 CA3 domain (aa317-400)	This study Addgene #217767
pSBbi-pur H-2Kb	pSBbi-pur	H2Kb (murine MHC-I allele)	Addgene #111623 (Gift from Yewdell laboratory)
pCMV(CAT)T7-SB100	pCMV	SB100X transposase	Addgene #34879 from [109]
pSbi-pur-KSR1-CA3-EGFP (C-KSR)	pSBbi-pur	Human KSR1 CA3 domain (aa317-400)-EGFP (C-KSR)	This study Addgene #217768
pSbi-pur-EGFP-KSR1-CA3 (N-KSR)	pSBbi-pur	EGFP-Human KSR1 CA3 domain (aa317-400) (N-KSR)	This study Addgene #217769
FcgRIIA-cmyc	pcDNA3	Myc-tagged Fc receptor FcgRIIa (FCGR2A)	Gift from S. Grinstein University of Toronto [111]
pGFP-PKC-C1(2)delta	pN2	GFP-N2-PKCdelta-C1(2) C1(2) domains of PKC delta (rat)	Addgene #21216 [58]
pYFP-DBD	pcDNA3	C1b diacylglycerol binding domain (DBD) of rat PKC beta II	Addgene #14874 [60]
pCMV6-XL5-mCherry-STIM1	pCMV6-XL5	mCherry inserted after the signal sequence of human STIM1	Gift from R. Lewis Stanford University [108]
p-mCherry-Sec22b	pCMV-mCherry-C1	rat Sec22b (ERS24) NM_001025686	Gift from T. Galli/ C. Vannier, Institute Jacques Monod Paris [110]
pTag-RFP-C		Cytosolic TagRFP	Evrogen FP141
pEGFP-N1		Vector for C-terminally tagged EGFP constructs	Clontech

pEGFP-C1		Vector for N-terminally tagged EGFP contructs	Clontech
pmRFP-N1		Vector for C-terminally tagged mRFP constructs	Addgene #54635 Gift from Robert Campbell, Michael Davidson, and Roger Tsien [107]

Supplementary Table S3. List of primers used in the study.

**Primer Name**	**F/R**	**Sequence**	**Purpose**
NheI_hKSR1_Ntag_F	For	CTAAGCTAGCGCCACCATGGGGAACCGCATTGATGACG	subcloning hKSR1(aa317-400) into pEGFP-N1
XhoI_hKSR1_Ntag_R	Rev	GATGCTCGAGCCGAGTTAGTGGCAGGAAGG	subcloning hKSR1(aa317-400) into pEGFP-N1
XhoI_hKSR1_Ctag_F	For	TACACTCGAGCTGGCGGTTCCTCTGGTGGTGGTGGTGCGGGGAACCGCATTGATGACG	subcloning hKSR1(aa317-400) into pEGFP-C1
KpnI_hKSR1_Ctag_R	Rev	CACGGGTACCTTACCGAGTTAGTGGCAGGAAGG	subcloning hKSR1(aa317-400) into pEGFP-C1
hKSR_CC359-362SS_F	For	GTGTCCCAGAAGAGCATGATATTTGGAGTGAAG	to introduce mutations (C359S, C362S) into KSR1
hKSR_CC359-362SS_R	Rev	GTGGGAGACCTGCGACAGCCAGGA	to introduce mutations (C359S, C362S) into KSR1
NheI_hPRKCZ_Ntag_F	For	CTAAGCTAGCGCCACCATGAGGAAGCTGTACCGTGCCAAC	subcloning hPRKCZ(aa123-193) into pEGFP-N1
XhoI_hPRKCZ_Ntag_R	Rev	TACACTCGAGAGGCTCTTGGGAAGGCATGAC	subcloning hPRKCZ(aa123-193) into pEGFP-N1
XhoI_hPRKCZ_Ctag_F	For	CTGACTCGAGCTGGCGGTTCCTCTGGTGGTGGTGGTGCGAGGAAGCTGTACCGTGCCAAC	subcloning hPRKCZ(aa123-193) into pEGFP-C1
KpnI_hPRKCZ_Ctag_R	Rev	GCGAGGTACCTTAAGGCTCTTGGGAAGGCATGAC	subcloning hPRKCZ(aa123-193) into pEGFP-C1
NheI_hSET_Ntag_F	For	ATTAGCTAGCGCCACCATGCAGAAGAGGTCAGAATTGATCG	subcloning hSET(aa70-226) into pEGFP-N1
XhoI_hSET_Ntag_R	Rev	GACTCTCGAGATCCATATCGGGAACCAAGTAG	subcloning hSET(aa70-226) into pEGFP-N1
XhoI_hSET_Ctag_F	For	AACGCTCGAGCTGGCGGTTCCTCTGGTGGTGGTGGTGCGCAGAAGAGGTCAGAATTGATCG	subcloning hSET(aa70-226) into pEGFP-C1
KpnI_hSET_Ctag_R	Rev	GACGGGTACCTTAATCCATATCGGGAACCAAGTAG	subcloning hSET(aa70-226) into pEGFP-C1
NheI-hPRKCZ-C20_F	For	TACAGCTAGCGCCACCATGGGTGACACAACGAGCACTTTC	subcloning PRKCZ-C20 (aa 405-646)fragment into pEGFP-N1
XhoI-hPRKCZ-C20_R	Rev	GATGCTCGAGCGACTCCTCGGTGGACAGC	subcloning PRKCZ-C20 (aa 405-646)fragment into pEGFP-N1
XhoI-hPRKCZ-C20_F	For	TACACTCGAGCTGGCGGTTCCTCTGGTGGTGGTGGTGCGGGTGACACAACGAGCACTTTC	subcloning PRKCZ-C20 (aa 405-646)fragment into pEGFP-C1
KpnI-hPRKCZ-C20_R	Rev	CAATGGTACCCGACTCCTCGGTGGACAGC	subcloning PRKCZ-C20 (aa 405-646)fragment into pEGFP-C1
XhoI-hKSR1-2X-F	For	GAACCTCGAGGGGAACCGCATTGATGACG	subcloning hKSR1(aa317-400) into pKSR1-EGFP
KpnI-hKSR1-2X-R	Rev	TACAGGTACCGACCGAGTTAGTGGCAGGAAGG	subcloning hKSR1(aa317-400) into pKSR1-EGFP
PRKCZ_C-C20-NES-F	For	GAGCTGGATGAGGCACCGGTCGCCACCGTGAGCAAGGGCGAGGAG	addition of MAPKK nuclear export signal to PRKCZ-C20-EGFP
PRKCZ_C-C20-NES-R	Rev	AAGCTCTTCCAACTTTTTCTGCAGAGCCATGGTGGCGACCGGTAG	addition of MAPKK nuclear export signal to PRKCZ-C20-EGFP
SET_N-C20-NES-F	For	GAGCTGGATGAGGCACCGGTCGCCACCCAGAAGAGGTCAGAATTGATCGCC	addition of MAPKK nuclear export signal to SET-EGFP
SET_N-C20-NES-R	Rev	AAGCTCTTCCAACTTTTTCTGCAGAGCCATGGTGGCGCTAGCGGA	addition of MAPKK nuclear export signal to SET-EGFP
pETM11-lin-F	For	ACTGAGATCCGGCTGCTAAC	linearization of vector pETM11-SUMO3-EGFP-sfGFP (Gibson assembly)

pETM11-lin-R	Rev	GGATCCACCGGTCTGTTGC	linearization of vector pETM11-SUMO3-EGFP-sfGFP (Gibson assembly)
KSR-EGFP-F	For	CAACAGACCGGTGGATCCGGGAACC GCATTGATGACG	KSR1 insert amplification to subclone into linearized pETM11-SUMO3-EGFP-sfGFP via Gibson assembly
EGFP-R	Rev	TAGCAGCCGGATCTCAGTTTACTTGT ACAGCTCGTCCATG	insert amplification to subclone EGFP-tagged probes into a linearized vector pETM11-SUMO3-EGFP-sfGFP via Gibson assembly
SfiI-KSR1-F	For	CATGGCCCACAGGGCCTGGGGAACC GCATTGATGACG	for subcloning KSR1 fragment onto SfiI sites of vector pETM11-SUMO3-SfiI-sfGFP
Sfi-KSR1-R	Rev	TACGGCCGATATGGCCTTACCGAGTT AGTGGCAGGAAGG	for subcloning KSR1 fragment onto SfiI sites of vector pETM11-SUMO3-SfiI-sfGFP
SfiI-probe-EGFP-F	For	CGTGGCCTCTGAGGCCTGAACCGTCA GATCCGCTAG	subloning C-terminally tagged KSR1 into pSBbi-pur
SfiI-probe-EGFP-R	Rev	TACGGCCTGACAGGCCTTACTTGTAC AGCTCGTCCATG	subloning C-terminally tagged KSR1 into pSBbi-pur
C-KSR-GSplus-mut-F	For	ggtggtggtgcgTCGACGGTACCGCGGGC C	deletes LELKLRILQS linker connecting KSR1 CA3 domain to EGFP (in C-KSR constructs) and adds GGSSGGGGA linker.
C-KSR-GSplus-mut-R	Rev	accagaggaaccgccCCGAGTTAGTGGCA GGAAGGATATTCTACAG	deletes LELKLRILQS linker connecting KSR1 CA3 domain to EGFP (in C-KSR constructs) and adds GGSSGGGGA linker.

**For = forward, Rev = reverse, F/R = forward or reverse*

Supplementary Table S4. Lipid detection parameters for lipidomic analysis

**Lipid class**	**Standard**	**Polarity**	**Mode**	**m/z ion**	**Collision energy eV**
Phosphatidylcholine $[M+H]^+$	DLPC	+	Product ion	184.07	30
Phosphatidylethanolamine [M+H]+	PE31:1	+	Neutral ion loss	141.02	20
Phosphatidylinositol [M-H]-	PI31:1	-	Product ion	241.01	44
Phosphatidyserine [M-H]-	PS31:1	-	Neutral ion loss	87.03	23
Cardiolipin [M-2H]2-	CL56:0	-	Product ion	Acyl chain	32
Ceramide	C17Cer	+	Product ion	264.34	25
Hexosylceramide	C8GC	+	Product ion	264.34	30
Sphingomyelin	C12SM	+	Product ion	184.07	26
