# Supplementary figures and images for: Development of Genetically Encoded Fluorescent KSR1-Based Probes to Track Ceramides during Phagocytosis
Source: Int J Mol Sci. 2024 Mar 5;25(5):2996. doi: 10.3390/ijms25052996 (PMC10932182; doi:10.3390/ijms25052996)

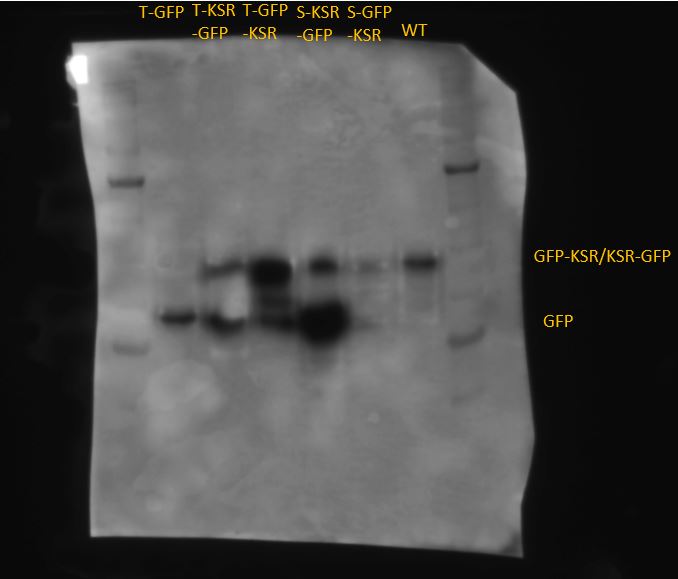

Supplement: Supplementary file 1 [file ijms-25-02996-s001.zip › Girik_WB/C-KSR-N-KSR_WB_results.JPG]

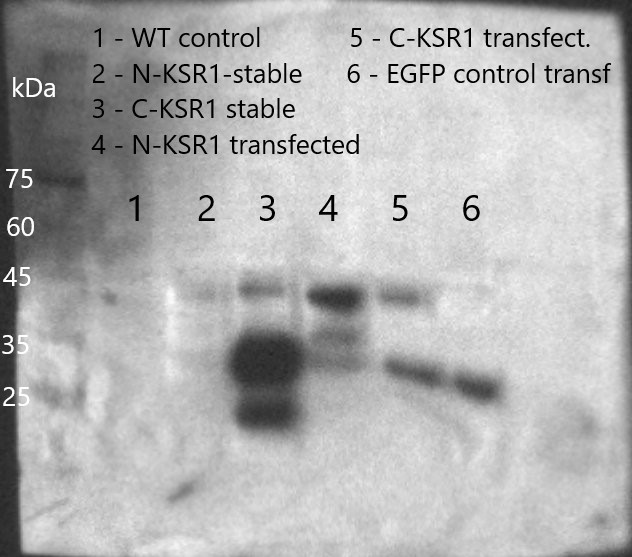

Supplement: Supplementary file 1 [file ijms-25-02996-s001.zip › Girik_WB/C-KSR1-N-KSR-WB-repeat-02-1s_annotated.jpg]

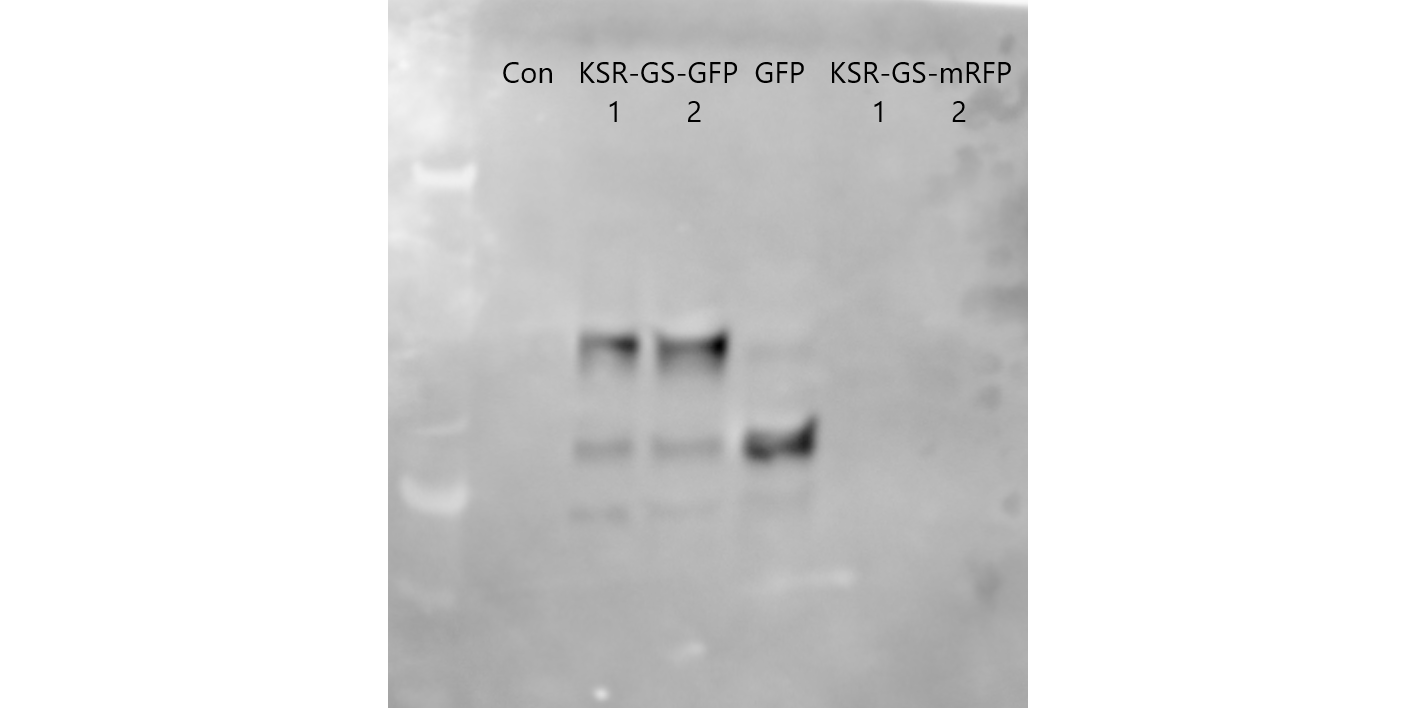

Supplement: Supplementary file 1 [file ijms-25-02996-s001.zip › Girik_WB/KSR-GS-WB_10s-annotated.png]

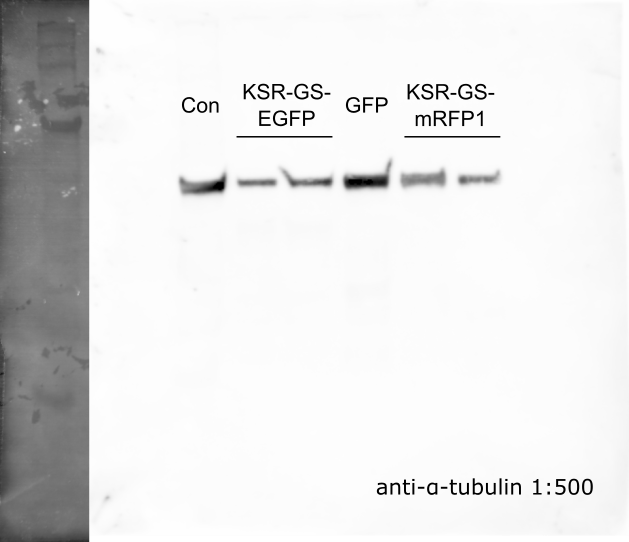

Supplement: Supplementary file 1 [file ijms-25-02996-s001.zip › Girik_WB/KSR-GS-WB_anti-tubulin-10s-annotated.png]
